# Supplementary material for: A new ornithurine from the Early Cretaceous of China sheds light on the evolution of early ecological and cranial diversity in birds
Source: PeerJ. 2016 Mar 15;4:e1765. doi: 10.7717/peerj.1765 (PMC4806634; doi:10.7717/peerj.1765)
Supplement: Table S1 [file peerj-04-1765-s003.docx]

| **Table S1 Measurements of *Changzuiornis angmi*, *Juehuaornis zhangi* and *Dingavis longimaxilla*  (in mm)** | | | |
| --- | --- | --- | --- |
| **Major elements** | ***Changzuiornis*(AGB5840)** | ***Juehuaornis* (SJG 00001)** | ***Dingavis* (V20284)** |
| Skull length | 65 | 63.3 | 58.64 |
| Synsacrum | 33* |  | 28.2 |
| Phgostyle | 9.1 |  | 7.8 |
| Humerus | 50.1/50.4 | 46.7/45.5 | 48.9/51* |
| Ulna | 53/52 | 55.5R | 44.9*/52* |
| Carpometacarpus | 31.3/30.3 | 31.1R | 30.4/29.9 |
| Alular metacarpal | 5.1/5.4 |  | 4.1/3.6* |
| Alular digit phalanx 1 | 11.7R | 11R | 12.4/12.5 |
| Alular digit phalanx 2 | 5.4R | 3.4R | 4.9/5 |
| Major digit phalanx 1 | 11.6/12.9 | 15.5R | 14/13.2 |
| Major digit phalanx 2 | 12.6/14.4 | 12.2R | 13.2/13.2 |
| Major digit phalanx 3 | 3.6R |  | 3.9/4 |
| Minor digit phalanx 1 | _ | 6.8 | 6.6/7.9 |
| Scapula | 46.4R | 41R | 38.4/36.4* |
| Pubis | 34.3R | 38.9*R | 42.7R |
| Femur | 29.4R | 33.3*R | 36*/36.1 |
| Tibiotarsus | 54/53.7 | 55.6R | 55.6/55.4 |
| Tarsometatarsus | 36.1/36 | 38.7R | 37.9/40.6 |
| * estimated; R/L**;** L, left; R, right. | |  |  |
